# Protective Efficacy Evaluation of Various Inactivated Vaccines Against the Newly Circulated Highly Pathogenic Avian Influenza Virus H5N1 of Clade 2.3.4.4b in Pekin Ducks

**DOI:** 10.3390/v18080891

**Published:** 2026-08-13

**Authors:** Eman Abd El Menum Shosha, Ibrahim Eldaghayes, Ahmed Abd-Elsamie H. Ali, Waleed Senosy Ali, Eman Hafez Elhayani

**Affiliations:** 1 Virology Department, Faculty of Veterinary Medicine, New Valley University, El-Khargia 72511, Egypt; 2 Department of Microbiology and Parasitology, Faculty of Veterinary Medicine, University of Tripoli, Tripoli 1300, Libya; 3 Virology Department, Faculty of Veterinary Medicine, Zagazig University, Zagazig 44519, Egypt; 4 Theriogenology Department, Faculty of Veterinary Medicine, New Valley University, El-Khargia 72511, Egypt

**Keywords:** HPAI, H5N1, inactivated H5 vaccines, efficacy, clade 2.3.4.4b, vaccination regimes

## Abstract

Highly pathogenic *avian influenza* (HPAI) *virus* H5N1of clade 2.3.4.4b has emerged as the predominant lineage circulating in poultry flocks worldwide, raising concerns regarding the protective efficacy of currently available commercial vaccines, particularly in domestic ducks, which play an important role in virus maintenance and transmission. Thus, this study evaluated the immunogenicity along with the protective efficacy of four inactivated H5 vaccines against a recently isolated local HPAI-H5N1 (Newvalley-3-H5N1-2024, clade 2.3.4.4b) strain in Pekin ducks in Egypt. A total of 150 seronegative ducks were divided into vaccinated and control groups (10 groups) and vaccinated at 10 days of age. At 31 days of age, the vaccinated and positive control groups were challenged using 10^6.5^ EID_50_/0.5 mL/duck with the local isolate (Newvalley-3-H5N1-2024) via the oculo-nasal route. The vaccine efficacy was assessed through clinical signs, survival rate, hemagglutination inhibition (HI) antibody titer, tracheal and cloacal viral shedding quantified by real-time RT-PCR, and histopathological examination of trachea, lung, pancreas, and brain tissues. Generally, all ducks vaccinated with the ValleyVac Avian Flu H5 plus and MEFLUVAC^TM^ H5 PLUS 8 showed a significantly higher survival rate (100%) at 10 days post-vaccination (DPV) than those in the positive control (66.7% mortality rate). In contrast, ducks exhibited mortality rates ranging from 6.7% in the SERVAC Flu H5N1 group to 13.4% in the Sinder Fluvac group. The ValleyVac Avian Flu H5 plus and MEFLUVAC™ H5 PLUS 8 vaccines induced the highest HI antibody titers at 7, 14, 21, and 28 DPV in both homologous and heterologous AIV antigens, resulting in a significant reduction in viral load among all vaccinated duck groups (*p*-value < 0.05) comparable to the positive control group. Conversely, the SERVAC Flu H5N1 and Sinder Fluvac vaccines provided partial protection, suboptimal immunogenicity at different time points, and elevated viral shedding. Histopathological findings in ValleyVac Avian Flu H5 plus and MEFLUVAC™ H5 PLUS 8 vaccines exhibited mild tissue alterations following AIV challenge. Marked pathological lesions were observed in the SERVAC Flu H5N1 and Sinder Fluvac vaccinated groups. Among tested vaccines, both ValleyVac Avian Flu H5 plus and MEFLUVAC^TM^ H5 PLUS 8 showed the highest level of protective efficacy against the circulating AIV strain compared with other commercial vaccines. This study highlights the need for continuous molecular surveillance, antigenic matching, and regular updating of vaccine seed strains to ensure efficient HPAI control in Egypt.

## 1. Introduction

In Egypt, the local poultry sector has faced various challenges due to the emergence of multiple viral pathogens spreading, leading to considerable economic losses through increased mortality, reduced productivity and high costs for disease prevention and control. RNA viruses have been considered a major threat due to their high genetic diversity, which facilitates rapid evolution and the emergence of multiple epidemics [1,2,3,4]. Since the late 2020s, the highly pathogenic avian influenza (HPAI) virus H5N1 belonging to clade 2.3.4.4b has caused widespread outbreaks in wild birds and domestic poultry across multiple regions of the world. Continuous molecular evolution and reassortment events have contributed to the emergence of numerous viral variants with altered epidemiological characteristics [5,6,7,8]. The HPAI of H5 subtype has caused significant economic harm to the poultry industry globally.

In Egypt, HPAI-H5N1 of clade 2.3.4.4b was first detected in migratory birds during late 2021, followed by outbreaks in domestic poultry populations in early 2022 [9]. Control of AIV in poultry mainly relies on the rapid eradication of infected flocks and the strategic use of vaccination programs employing commercial inactivated vaccines that are genetically and antigenically similar to circulating strains in the field [10,11]. Following the entry of HPAI-H5N8 of clade 2.3.4.4b, Egypt updated its H5 vaccination regime in 2017 to improve protection against the newly emerging viruses [12]. However, the subsequent entry of HPAI-H5N1 of clade 2.3.4.4b into the local poultry sector in Egypt further complicated the epidemiological situation because of the simultaneous circulation of different H5 lineages [13]. Moreover, HPAI-H5N1 of clade 2.2.1 was introduced into Egypt in 2006 and became endemic in poultry by 2008 [14]. Ongoing viral evolution led to the appearance of the clade 2.2.1.2 lineage, which was associated with severe economic losses in the poultry industry and numerous zoonotic infections in humans [15,16]. Although H5N1of clade 2.2.1.2 has not been detected since 2017, this is likely due to the implementation of locally developed inactivated H5N1vaccines with improved antigenic matching and its replacement with HPAI-H5N8 of clade 2.3.4.4b. The potential re-emergence of this lineage cannot be excluded and remains a significant concern for disease control [17].

Multiple genotypes of HPAI-H5N1 belonging to clade 2.3.4.4b have emerged and spread extensively among wild birds since late 2020, with subsequent detection in numerous countries across Africa, Asia, Europe, and North America [18,19,20,21]. Currently, the HPAI-H5N1 of clade 2.3.4.4b is the predominant AIV lineage responsible for AIV outbreaks worldwide, and has existed in a wide range of host species, indicating its expanding host range and increased zoonotic potential [22]. In Egypt, AIV control relies on different vaccination programs, where inactivated vaccines are employed as a preventive strategy to minimize mortality rates and viral transmission among poultry flocks. In particular, the effectiveness of these vaccination programs depends on the ability of the vaccines to suppress viral replication and shedding, thereby limiting flock-to-flock spread and reducing the emergence of new reassortant viruses [23,24,25]. However, the continuous genetic evolution and antigenic divergence of the circulating H5 viruses have led to a distinct antigenic mismatch between the vaccine strain and the circulating field isolate, and have diminished the effectiveness of traditional vaccination strategies, resulting in outbreaks and production losses even among vaccinated flocks. Therefore, the continued occurrence of H5N1 infections has highlighted the need for continuous monitoring and regular evaluation of commercially used vaccines against newly emerging field strains to ensure adequate antigenic matching and to support the development of optimized vaccination strategies [26,27,28]. Within Egypt’s multi-species poultry production landscape, domestic waterfowl—particularly ducks—occupy a vital epidemiological role in the persistence and dissemination of *avian influenza viruses*. Unlike chickens, which typically suffer rapid, high mortality following HPAI infection, ducks frequently display variable clinical outcomes ranging from mild or subclinical disease to prolonged asymptomatic infection. Consequently, infected ducks act as silent carriers and viral amplifiers, continuously shedding high titers of AIV through both mucosal respiratory and digestive routes into shared aquatic environments, drainage canals, and live bird market (LBM) networks [29,30,31,32,33]. This continuous shedding without obvious disease signs facilitates covert viral circulation, accelerates antigenic drift under field conditions, contributing to persistence, and poses an ongoing spillover risk to poultry flocks and humans. In Egypt, the commercial and backyard duck production represents an important sector of the local poultry industry, and ducks are often reared in close proximity to chickens. Thus, effective vaccination of ducks is essential not only to reduce mortality but also to limit viral shedding, interrupt transmission, and decrease the risk of emergence and dissemination of antigenically divergent viruses. Therefore, evaluating commercial H5 vaccine efficacy in ducks—specifically focusing on the suppression of mucosal replication and shedding—is critical for establishing effective control strategies that target the primary source of viral maintenance in Egypt [34,35,36]. Currently, many avian influenza vaccines used in Egyptian poultry production are generated through reverse-genetics (rg) technology. These recombinant vaccine candidates typically express the surface glycoproteins, the hemagglutinin (HA) and neuraminidase (NA) genes, from the targeted H5 field isolate together with the six internal genes [37,38]. Consequently, this study evaluated the immune response and protective efficacy of various vaccination regimens using commercial inactivated vaccines against recently circulating local HPAI-H5N1 by examining clinical signs, pathological findings, HI tests, and viral shedding evaluation. This experimental vaccination protocol was applied in Pekin ducks to assess its effectiveness against local strain AIV challenge and to optimize vaccination strategies.

## 2. Materials and Methods

### 2.1. Ethical Statement and Care of the Experimental Birds

Animal ethics and welfare standards were strictly maintained throughout the study. The study’s experimental protocols were approved by the Ethics Committee within the Faculty of Veterinary Medicine at New Valley University, Egypt (Approval No. 02/2/6-2024/2), ensuring proper bird care and minimizing distress. All experimental procedures were performed within a biosafety level 3 (BSL-3) laboratory. Following the HPAI challenge, birds were monitored at least twice daily for clinical signs and welfare status throughout the experimental period. A predefined humane endpoint protocol, approved by New Valley University’s Local Ethics Commission for Animal Experiments, was implemented. Birds were humanely euthanized if they exhibited one or more of the following criteria persisting for 24–48 h: severe depression or unresponsiveness, inability to reach feed or water, severe respiratory distress (gasping or labored breathing), persistent neurological signs (e.g., torticollis, paralysis, inability to stand or maintain balance), prolonged recumbency, or a moribund condition indicating that recovery was unlikely. Euthanasia was performed by inhalation of isoflurane (>5%) for approximately 1 min until cessation of respiration, followed by cervical dislocation to confirm death. All surviving birds were euthanized at the end of the experiment using the same approved procedure. Carcasses were subsequently disinfected in 10% formalin for 2 h before disposal by incineration in accordance with institutional biosafety regulations.

### 2.2. Commercial Vaccines and Virus Strains

Four commercial inactivated avian influenza vaccines including, ValleyVac Avian Flu H5 plus commercial bivalent vaccine (Vaccine Valley, Giza, Egypt)^®^, SERVAC Flu H5N1 monovalent vaccine (Veterinary Serum and Vaccine Research Institute, Cairo, Egypt)^®^, MEFLUVAC^TM^ H5 PLUS 8^®^ (MEVAC, Cairo, Egypt)^®^ is a reverse genetically engineered inactivated trivalent vaccine, and Sinder Fluvac bivalent H5N1 inactivated vaccine (Shandong Sinder Animal Vaccine Co., Ltd., Zhucheng, China)^®^ were used in this study (Table 1). These four vaccines were selected as representative candidates of the primary commercial formulation strategies in Egypt, encompassing monovalent, bivalent, and trivalent reverse-genetics (RG) or wild-type strains from public, local commercial, and imported suppliers widely used in duck production in Egypt. ValleyVac Avian Flu H5 Plus contains reverse-genetics (RG)-derived H5N1 and H5N8 reassortant strains, MEFLUVAC™ H5 PLUS 8 contains RG-derived H5N1 and H5N8 vaccine seed strains, and Sinder Fluvac contains the internationally recognized Re-6 and Re-8 RG-derived H5 vaccine strains. In contrast, SERVAC Flu H5N1 is produced from an inactivated Egyptian H5N1 field isolate (A/Chicken/Egypt/M2583D/2010; clade 2.2.1.1) (Table 1). All the respective inactivated vaccines were administered to ducks via the subcutaneous (S/C) route at the recommended dose of 0.5 mL/bird in the lower part of the neck at the age of 10 days according to the manufacturer’s guidelines. The challenge virus used was a HPAI strain, Newvalley-3-H5N1-2024, isolated in 2024 (GenBank accession no.: PX062294, clade 2.3.4.4b). The challenge virus was propagated and titrated using embryonated chicken eggs (ECEs) according to the method described by Reed and Muench [38,39], which utilizes the egg infective dose at 50% (EID_50_).

### 2.3. Experimental Design and Duck Challenge

One-day-old healthy Pekin ducks (n = 150), negative for specific antibodies, were purchased from a commercial hatchery in El-Sharquia governorate, Egypt. The ducks were randomly divided into 10 groups (15 ducks per group) within separate isolators under biosecurity conditions. All tested ducks were supplied with feed and water ad libitum during the entire experiment. In addition, vaccination regimes were administered at 10 days of age (Table 2). At 31 days of age, the ducks were challenged with the NewValley-3-H5N1-2024 (GenBank accession no.: PX062294, clade 2.3.4.4b, and isolated from New Valley governorate) with 10^6.2^ EID_50_/mL via the oculo-nasal route with a dose of 0.5 mL per duck. Importantly, tracheal and cloacal swabs were collected at 1, 3, 5, and 7 days post-challenge (DPC) from challenged ducks for virus shedding quantification. The collected swab specimens were suspended in ice-cold phosphate-buffered saline (PBS, 1 mL) and preserved at −80 °C until molecular analysis using quantitative real-time RT–PCR. All ducks were monitored twice daily, with clinical manifestations, mortality rates, and post-mortem lesion detection recorded in euthanized and dead birds. Virus back-titration was performed in 9–11-day-old SPF embryonated chicken eggs immediately after viral inoculation to verify the challenge dose. The back-titration confirmed a stock viral concentration of 10^6.5^ EID_50_/mL, yielding an actual delivered dose of 10^6.2^ EID_50_ per duck (0.5 mL/per bird). The ducks were obtained from a non-vaccinated farm and were serologically tested upon entering the biosecurity level-3-enhanced (BSL-3E) facility located at the Central Laboratory for Evaluation of Veterinary Biologics (CLEVB), Cairo, Egypt. The detection of antibodies in serum samples was performed using the hemagglutination inhibition (HI) test, which included 1% chicken red blood cells (RBCs), confirming that all ducks were serologically negative for AIV-H5. Additionally, the trachea, pancreas, lungs, and brain were collected for histopathological investigation (3 ducks/group at 10 DPC) [40]. The unvaccinated–challenged (Group 9) and unvaccinated/unchallenged ducks (Group 10) were kept as control groups throughout the experiment. The ducks in the negative control (Group 10) were sham-challenged with PBS through the oculo-nasal route at the same time point.

### 2.4. Serological Monitoring

To evaluate antibody kinetics without confounding post-challenge parameters, a parallel-cohort study design was implemented. Vaccinated non-challenged groups (G2, G4, G6, and G8) were divided into parallel pairs receiving identical vaccine administration. Groups (G2), (G4), (G6), and (G8) served as parallel non-challenged serological tracking groups for the challenged groups (G1, G3, G5, and G7) to trace weekly HI antibody kinetics at 7, 14, 21, and 28 DPV. Blood samples from the brachial (wing) vein were collected weekly from vaccinated groups (G2, G4, G6, and G8) for antibody titer detection at 7, 14, 21, and 28 DPV. Then, the collected samples were centrifuged at 5000× *g* for 10 min to separate the serum. The commercial vaccines’ immunogenicity was evaluated by determining anti-hemagglutinin antibody titers using hemagglutination inhibition (HI) assay in U-bottom 96-well microtiter plates using 4 HA units (HAU). The HI assay was conducted following the standard guidelines mentioned by the World Organization for Animal Health (WOAH, previously known as OIE) [41]. Two antigens were used for serological assessments: a heterologous reference H5 antigen (GD-Netherlands) and a homologous antigen prepared from a local challenge strain (Newvalley-3-H5N1-2024, belonging to clade 2.3.4.4b). Furthermore, a standardized heterologous reference H5 antigen commercially manufactured by Royal GD Animal Health [Deventer, The Netherlands; H5N8 inactivated HI antigen (VLDIA 354 HAG), influenza virus A/Chicken/Netherlands/EMC-3/2014, HPAI H5N8, HA clade 2.3.4.4c]. Both homologous and heterologous antigens were used to evaluate the specificity of antibody responses induced by vaccination. Specifically, the heterologous antigen (clade 2.3.4.4c) was utilized to measure cross-reactive humoral responses, while the homologous challenge strain antigen (clade 2.3.4.4b) was used to measure strain-specific neutralization and assess protective antibody thresholds against the contemporary circulating virus. Comparing titers between both antigens allowed for a clear differentiation between broad cross-reactive antibody responses and strain-specific homologous antibody responses. In accordance with the WOAH terrestrial manual, HI antibody titers of ≥1:32 (≥5 log_2_) detected in at least 80% of vaccinated birds are considered indicative of the seroprotective threshold for preventing mortality [41,42].

### 2.5. Quantification of Viral Shedding

In particular, tracheal and cloacal swabs from all challenged ducks were collected (n = 5 for each swab/group) in each experimentally challenged group on days 1, 3, 5, and 7 (DPC) to quantify viral shedding. Viral RNA was extracted from the collected swabs using the QIAamp Viral RNA Mini Kit (Qiagen, Hilden, Germany) following the manufacturer’s instructions. Then, the matrix gene fragment was subsequently amplified using a Stratagene MX3005P real-time PCR system, with a quantitative real-time reverse transcriptase PCR (qRT-PCR) Kit (QuantiTect^®^ probe RT-PCR kit, Qiagen, Hilden, Germany). Specific oligonucleotide primers and probes were used as described by [43], and all thermal cycling conditions were performed according to their recommendations. The vaccine efficacy, expressed as the percentage of protection, was calculated according to the following formula [44]: number of survivors ÷ total number of challenged birds × 100. Importantly, the quantification of AIV shedding titers was determined by a standard curve established using tenfold serial dilutions of a reference virus inoculated in specific pathogen-free embryonated chicken eggs (SPF-ECEs). The viral loads in collected specimens were subsequently calculated by interpolating the Ct values against the obtained standard curve, allowing accurate differentiation between vaccine-derived and challenge AIV strains [17]. Results are expressed as log_10_-AIV RNA copy numbers per milliliter (mL) of sample.

$$\mathrm{Protection}\;\mathrm{percentage}=\left(\frac{\mathrm{Number}\;\mathrm{of}\;\mathrm{survivals}}{\mathrm{Total}\;\mathrm{number}\;\mathrm{of}\;\mathrm{challenged}\;\mathrm{birds}}\times 100\right)$$

### 2.6. Pathological Examination

At 10 days post-challenge, three ducks, randomly selected, from each experimental group were euthanized and subjected to gross pathological examination. The trachea, lung, pancreas, and brain tissue specimens were collected for subsequent histopathological evaluation. Tissue specimens were fixed in neutral buffered formalin (10%), then processed and embedded in paraffin wax. These samples were sectioned at 4 µm thickness and stained with hematoxylin and eosin (H&E). A microscopic examination was performed using a light microscope [38]. Briefly, semi-quantitative histopathological scoring was estimated according to a previously reported method [45] by a veterinary pathologist who was blinded to the experimental treatment groups. Organs were evaluated for different pathological parameters (Table S1), and lesion severity was graded according to their severity on a four-point scale: 0 (normal), 1 (mild), 2 (moderate), and 3 (severe) [46,47]. Regarding the histopathological scoring, five randomly selected microscopic fields were examined for each specimen using 10× and 20× magnification by light microscope (Nikon-Eclipse 50i, Nikon Instruments Inc., Melville, NY, USA). The mean lesion scores and standard deviation were calculated for each group, and the summarized data are presented in Table S1. Histopathological examination was only conducted on ducks from the challenged groups (G1, G3, G5, and G7), together with the positive control (G9) and negative control (G10). The vaccinated, non-challenged groups (G2, G4, G6, and G8) were reserved only for serological assessment and were therefore excluded from histopathological examination. These birds were not exposed to the challenge virus, and they remained clinically normal throughout the study.

### 2.7. Statistical Analysis

Data are expressed as mean ± standard deviation (SD). The normality of the data distributions was evaluated using the Shapiro–Wilk normality test. Differences were subsequently analyzed among experimental groups in histopathological scores, HI antibody titers, and quantitative AIV shedding titers using the nonparametric Kruskal–Wallis test. When significant differences were indicated, Tukey’s honestly significant difference (HSD) post hoc multiple comparisons test was performed to detect pairwise differences among groups. Semiquantitative histopathological scores were generated and visualized using the ggplot2 package (version 4.0.3). Furthermore, a *p*-value (≤0.05) was considered statistically significant for all comparisons. Moreover, differences (*p*-values) in survival rate among the groups were determined using a log-rank test based on the Kaplan–Meier survival method. The detection limit corresponding to an infectivity titer was established at 0.7 log_10_ EID_50_/mL.

## 3. Results

### 3.1. Clinical Protection, Clinical Observations, Mortality Rate, and PM Lesions

**Figure 1:**
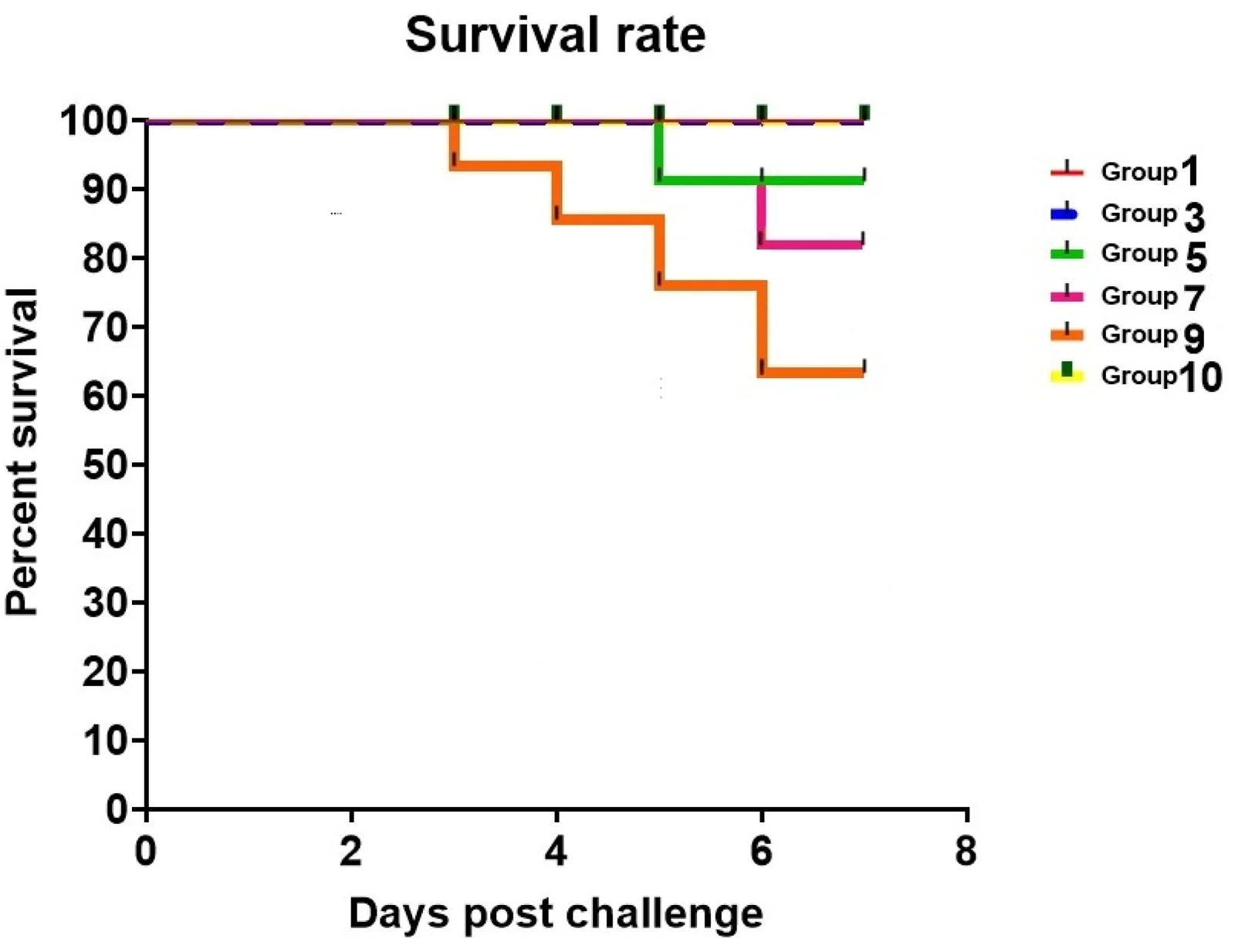
Kaplan–Meier survival curve of challenged and control ducks after immunization with four H5-inactivated commercial vaccines. (G1) ducks vaccinated with ValleyVac Avian Flu H5 plus, (G3) ducks vaccinated with MEFLUVAC^TM^ H5 PLUS 8, (G5) ducks vaccinated with SERVAC Flu H5N1, (G7) ducks vaccinated with Sinder Fluvac, (G9) positive control, and (G10) negative control.

Viral back-titration conducted in SPF embryonated chicken eggs immediately post-inoculation confirmed that the stock viral concentration was 10^6.5^ EID_50_/mL, corresponding to an actual delivered challenge dose of 10^6.2^ EID_50_ per duck in a 0.5 mL volume. All ducks were confirmed seronegative for AI virus-specific antibodies as determined by the HI before vaccination. No local reactions at the injection site or systemic clinical abnormalities were observed in any of the vaccinated ducks following administration. Seroconversion among vaccinated groups varied according to the vaccine formulation and serological assay used. Ducks in the nonvaccinated control group remained seronegative throughout the study period across all diagnostic tests. Experimental AI H5N1challenge on the third day resulted in 6.7%, 13.4%, and 66.7% mortality rates in the vaccinated (G5) and (G7) groups and the positive control (G9) group, respectively, with deaths occurring between days 3 and 6 post-challenge. Concerning the nonvaccinated challenged group (G9), clinical symptoms were observed three days post-infection (DPI) onwards. At the beginning of the observation period, one duck displayed mild depression, while several others developed light green, watery droppings. By 4 dpi, the clinical status of four ducks had worsened, and they were in poor general condition. In addition, these ducks exhibited reduced feed intake, depression, lethargy, light green watery diarrhea, ataxia, torticollis, and feather ruffling, which manifested from 4 DPC to 10 DPC. The birds in the negative control group (G10) remained clinically normal, with no signs consistent with AIV infection throughout the observation period. All ducks vaccinated with the ValleyVac Avian Flu H5 plus and MEFLUVAC^TM^ H5 PLUS 8 (G1, G3) survived up to 6 days post-infection and did not display any clinical manifestations of AIV in the surviving birds compared with those in (G9). In contrast, ducks that received the other commercial vaccines exhibited mortality following challenge, with mortality rates ranging from 6.7% in the SERVAC Flu H5N1 group to 13.4% in the Sinder Fluvac group, with mild signs in some vaccinated ducks. However, the severity of clinical manifestations in these vaccinated groups was milder than that observed in the nonvaccinated challenged control positive group, which showed a significantly higher mortality rate of 66.7%. Notably, the (G1) and (G3) groups showed significantly higher survival rates (100%) in the vaccinated birds with ValleyVac Avian Flu H5 plus and MEFLUVAC^TM^ H5 PLUS 8 at 10 days post-vaccination (no evidence of mortality), demonstrating a high level of protective efficacy under the study conditions. Furthermore, the survival rates in (G5) and (G7) were significantly improved (93.3% and 86.6%, respectively) compared with the positive control (33.3%) (Figure 1). Postmortem examination of dead ducks in the positive control group (G9) revealed pathological changes at 3–6 DPC, including carcass dehydration, petechial to ecchymotic hemorrhages in skeletal muscles, hyperemic and edematous tracheal mucosa, congested lungs, hemorrhagic tracheitis with associated pneumonitis, mild hemorrhages at the proventricular tips, and multifocal pancreatic necrosis. Similarly, other dead challenged ducks (G5 and G7) showed very mild lesions compared with those in (G9).

### 3.2. Humoral Immune Response in Ducks Induced by Four Commercial Vaccines

**Figure 2:**
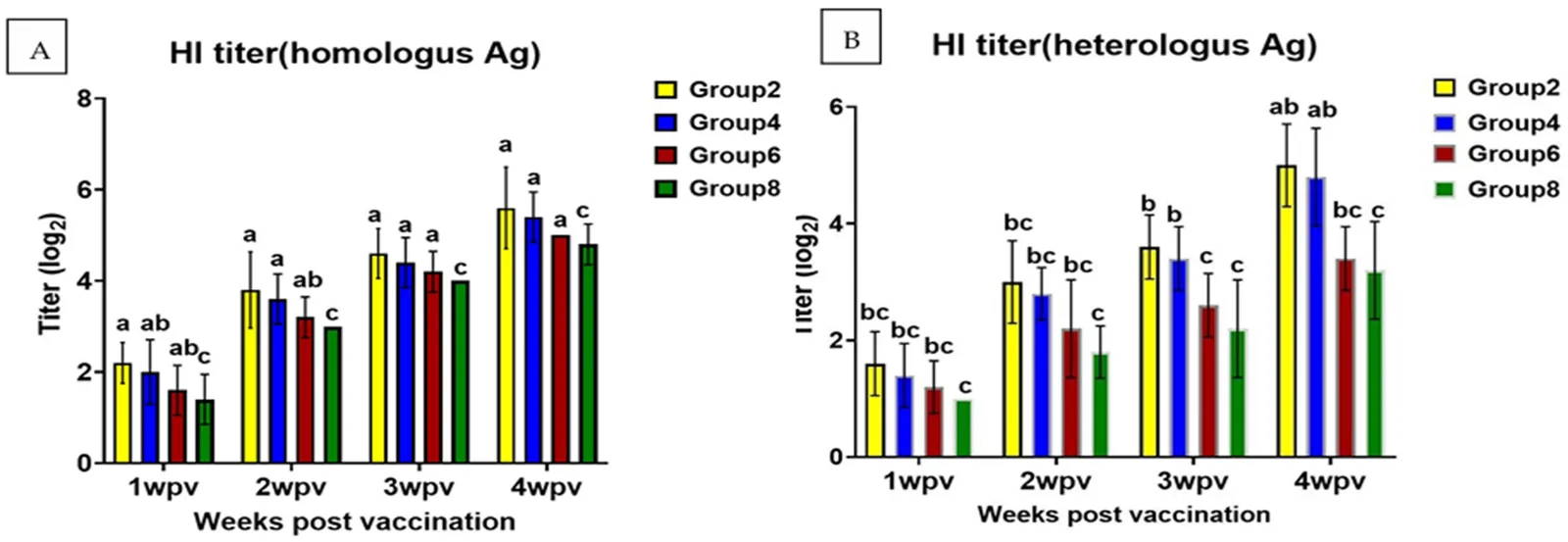
Hemagglutination inhibition antibody titers (log_2_) line graph by HI assay in ducks (G2, G4, G6, and G8) vaccinated with AIV commercial vaccines via the homologous challenge virus antigen (Newvalley-3-H5N1-2024 of clade 2.3.4.4b) (**A**). Heterologous reference H5 antigen (**B**) at 7, 14, 21, and 28 DPV. Data are expressed as group mean ± SD titer from five ducks per group. Statistical Variations between groups were determined via Tukey’s post hoc multiple comparisons test, with significance statistically established at *p*-value ≤ 0.05. Different lowercase letters above the bars indicate statistically significant differences between groups within the same vaccination week (*p* ≤ 0.05), whereas bars sharing at least one common lowercase letter are not significantly different. Statistical comparisons were performed separately at each sampling time point.

The immunogenicity of the four commercial vaccines in Pekin ducks was evaluated by detecting anti-hemagglutinin titers at 7, 14, 21, and 28 DPV (Figure 2A,B, Figure S1, Supplementary Material). Concerning homologous AIV antigens, early HI antibody responses were detected at 7 DPV in both ValleyVac Avian Flu H5 plus (G2) and MEFLUVAC^TM^ H5 PLUS 8 (G4), with titers of 2.2 ± 0.07 log_2_ and 2 ± 0.12 log_2_, respectively. On the same day, the SERVAC Flu H5N1 group (G6) and the Sinder Fluvac (G8) exhibited 1.6 ± 0.08 log_2_ and 1.4 ± 0.14 log_2_, respectively. By day 14, PV, the (G2), (G4), (G6), and (G8) groups showed 3.8 ± 0.05 log_2_, 3.6 ± 0.09 log_2_, 3.2 ± 0.21 log_2_, and 3 ± 0.23 log_2_, respectively. The HI titers on days 21 and 28 were higher than those on the previous days (Figure 2A, Table S2, Supplementary Material). At 21PV, the HI titer was 4.6 ± 0.14, 4.4 ± 0.18, 4.2 ± 0.20, and 4 ± 0.28 for the (G2), (G4), (G6), and (G8) groups, respectively. Surprisingly, post hoc comparisons using Tukey’s HSD test demonstrated that vaccinated groups receiving both ValleyVac Avian Flu H5 plus and MEFLUVAC^TM^ H5 PLUS 8 vaccines at 28 DPV induced a noticeably higher HI antibody titer of 5.6 ± 0.08 and 5.4 ± 0.14, respectively, which was significantly different (*p* < 0.05) compared with previously vaccinated groups. Likewise, SERVAC Flu H5N1 and the Sinder Fluvac vaccinated groups exhibited slightly high HI antibody titers of 5 ± 0.21 and 4.8 ± 0.24, *p* < 0.05, respectively. Furthermore, tests conducted using AIV heterologous reference H5 antigen showed HI titers of 1.6 ± 0.08 log_2_, 1.4 ± 0.13 log_2_, 1.2 ± 0.23 log_2_, and 1 ± 0.28 log_2_ (*p* < 0.05) in the (G2), (G4), (G6), and (G8) groups at day 7 PV, respectively (Figure 2B, Table S3, Supplementary Material). Meanwhile, all the vaccinated duck groups that evaluated HI titers on the 14th day were 3 ± 0.2 log_2_, 2.8 ± 0.12 log_2_, 2.2 ± 0.15 log_2_, and 1.8 ± 0.14 log_2_ (*p* < 0.05) in the (G2), (G4), (G6), and (G8) groups, respectively. By day 21 PV, the (G2) and (G4) groups exhibited an increase in HI titer of 3.6± 0.04 log_2_ and 3.4 ± 0.06 log_2_ compared to the (G6) and (G8) groups, which showed titers of 2.6 ± 0.11 log_2_ and 2.2 ± 0.15 log_2_. Finally, by day 28, the (G2) and (G4) groups displayed significantly higher HI antibody levels (*p* < 0.0001) of 5 ± 0.07 log_2_ and 4.8 ± 0.08 log_2_ compared with the (G6) and (G8) groups, which exhibited 3.4 ± 0.1 log_2_ and 3.2 ± 0.18 log_2_, respectively. Overall, the ValleyVac Avian Flu H5 plus and MEFLUVAC^TM^ H5 PLUS 8 commercial vaccines demonstrated the most robust and earliest rapid immune responses, whereas SERVAC Flu H5N1 and the Sinder Fluvac vaccines exhibited suboptimal immune responses (Figure 2A,B). Furthermore, the antibody HI titers were higher on days 7, 14, 21, and 28 DPV when evaluated using homologous local H5N1 antigen against the used vaccines than when evaluated using heterologous reference H5 antigen.

### 3.3. Viral Shedding by qRT-PCR in Challenged Ducks

**Figure 3:**
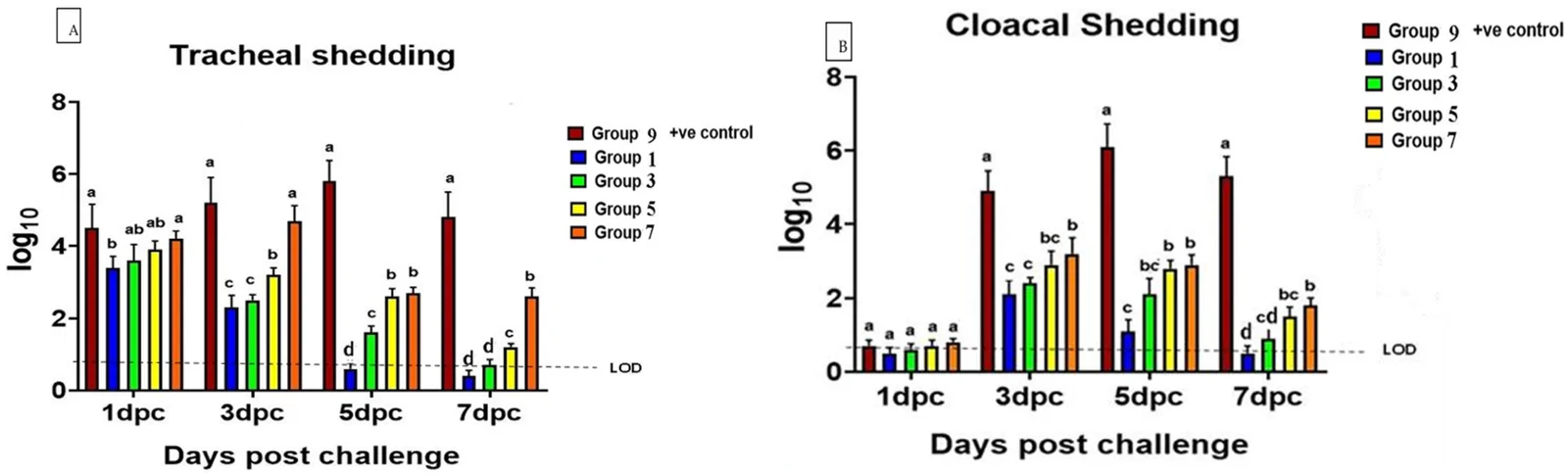
Viral shedding in tracheal and cloacal swabs collected in immunized ducks at 1, 3, 5, and 7 DPC. Panels represent viral shedding from (**A**) tracheal swabs and (**B**) cloacal swabs. Vaccinated ducks with commercial inactivated vaccines were challenged with 10^6. 2^EID_50_/0.5 mL dose/duck of the HPAI Newvalley-3-H5N1-2024 (clade 2.3.4.4b strain) via the oculo-nasal route. The horizontal dotted line indicates the limit of detection (LoD) at a titer of 0.7 log_10_ EID_50_. Data are expressed as group mean ± SD titer from five ducks per group. Statistical variations between groups were determined via Tukey’s post hoc multiple comparisons test, with significance statistically established at *p*-value ≤ 0.05. Among all vaccinated groups, the group vaccinated with ValleyVac Avian Flu H5 Plus vaccine exhibited the highest reduction in viral shedding, followed by the MEFLUVAC™ H5 PLUS 8 vaccine, which was comparable to that of the positive control group across all time points. Conversely, ducks vaccinated with the Sinder Fluvac vaccine showed the highest levels of viral shedding (lowest protection level). Different lowercase letters above the bars indicate statistically significant differences between groups within the same vaccination week (*p* ≤ 0.05), whereas bars sharing at least one common lowercase letter are not significantly different. Statistical comparisons were performed separately at each sampling time point.

To investigate the efficacy of AIV commercial vaccines in controlling viral shedding after challenging with the Newvalley-3-H5N1-2024, clade 2.3.4.4b strain, we measured virus shedding in tracheal (Figure 3A) and cloacal (Figure 3B) swabs significantly in all groups at days 1, 3, 5, and 7 DPC. Statistically, the shedding titers at 3, 5, and 7 DPC revealed a significant viral load reduction among all vaccinated duck groups (*p*-value < 0.05) compared with the positive control group. In the unvaccinated challenged (control positive) group, the highest shedding titers were detected in both tracheal (4.5 ± 0.32, 5.2 ± 0.45, 5.8 ± 0.48, 4.8 ± 0.35 log_10_ EID_50_/mL) and cloacal (0.7 ± 0.17, 4.9 ± 0.033, 6.1 ± 0.48, 5.3 ± 0.40 log_10_ EID_50_/mL) swabs on days 1, 3, 5, and 7 post-challenges, respectively. In tracheal swabs, there was a significant reduction in mean viral titers (3.4 ± 0.13, 2.3 ± 0.12 log_10_ EID_50_/mL) on 1 and 3 DPC, respectively, in the ValleyVac Avian Flu H5 plus group (*p*-value < 0.05). In the same group, the mean viral titers of cloacal swabs were 2.1 ± 0.18 and 1.1 ± 0.14 log_10_ EID_50_/mL on 3 and 5 DPC, respectively. Likewise, in the MEFLUVAC^TM^ H5 PLUS 8 group, the mean viral titers of tracheal swabs were 3.6 ± 0.19, 2.5 ± 0.21, and 1.6 ± 0.09 log_10_ EID_50_/mL on 1, 3, and 5 DPC (*p*-value < 0.05), respectively. Meanwhile, the mean viral titers of cloacal swabs of the same group were 2.4 ± 0.27, 2.1 ± 0.29, and 0.9 ± 0.11 log_10_ EID_50_/mL on 3, 5, and 7 DPC, respectively. Furthermore, the mean viral titers of tracheal swabs in the SERVAC Flu H5N1 vaccinated group were 3.9 ± 0.33, 3.2 ± 0.28, 2.6 ± 0.24, and 1.2 ± 0.22 log_10_ EID_50_/mL on 1, 3,5, and 7 DPC, respectively. The mean viral titers of cloacal swabs of the same group were 2.9 ± 0.29, 2.8 ± 0.29, and 1.5 ± 0.17 log_10_ EID_50_/mL on 3, 5, and 7 DPC, respectively. Concerning the Sinder Fluvac vaccinated group, the mean viral titers of tracheal swabs were 4.2 ± 0.28, 4.7 ± 0.31, 2.7 ± 0.21, and 2.6 ± 0.21 log_10_ EID_50_/mL on 1, 3,5, and 7 DPC, respectively. The mean viral titers of cloacal swabs of the same group were 0.8 ± 0.13, 3.2 ± 0.34, 2.9 ± 0.25, and 1.8 ± 0.21 log_10_ EID_50_/mL on 1, 3, 5, and 7 DPC, respectively. No AIV was detected in the trachea on days 5 and 7 DPC or cloacal swabs on days 1 and 7 DPC collected from ducks vaccinated with ValleyVac Avian Flu H5 plus vaccine. Similarly, no AIV was detected in the trachea on 7 DPC or cloacal swabs on 1 DPC in the case of MEFLUVAC^TM^ H5 PLUS 8 vaccine. No AIV was detected in cloacal swabs on day 1 post-challenge in the SERVAC Flu H5N1 and Sinder Fluvac vaccinated groups. Generally, a higher viral load was observed in the vaccinated groups in tracheal swabs than in cloacal swabs at both 1 and 3 DPC, then decreased at 5 and 7 DPC (*p*-value < 0.05).

### 3.4. Histopathological Examination

**Figure 4:**
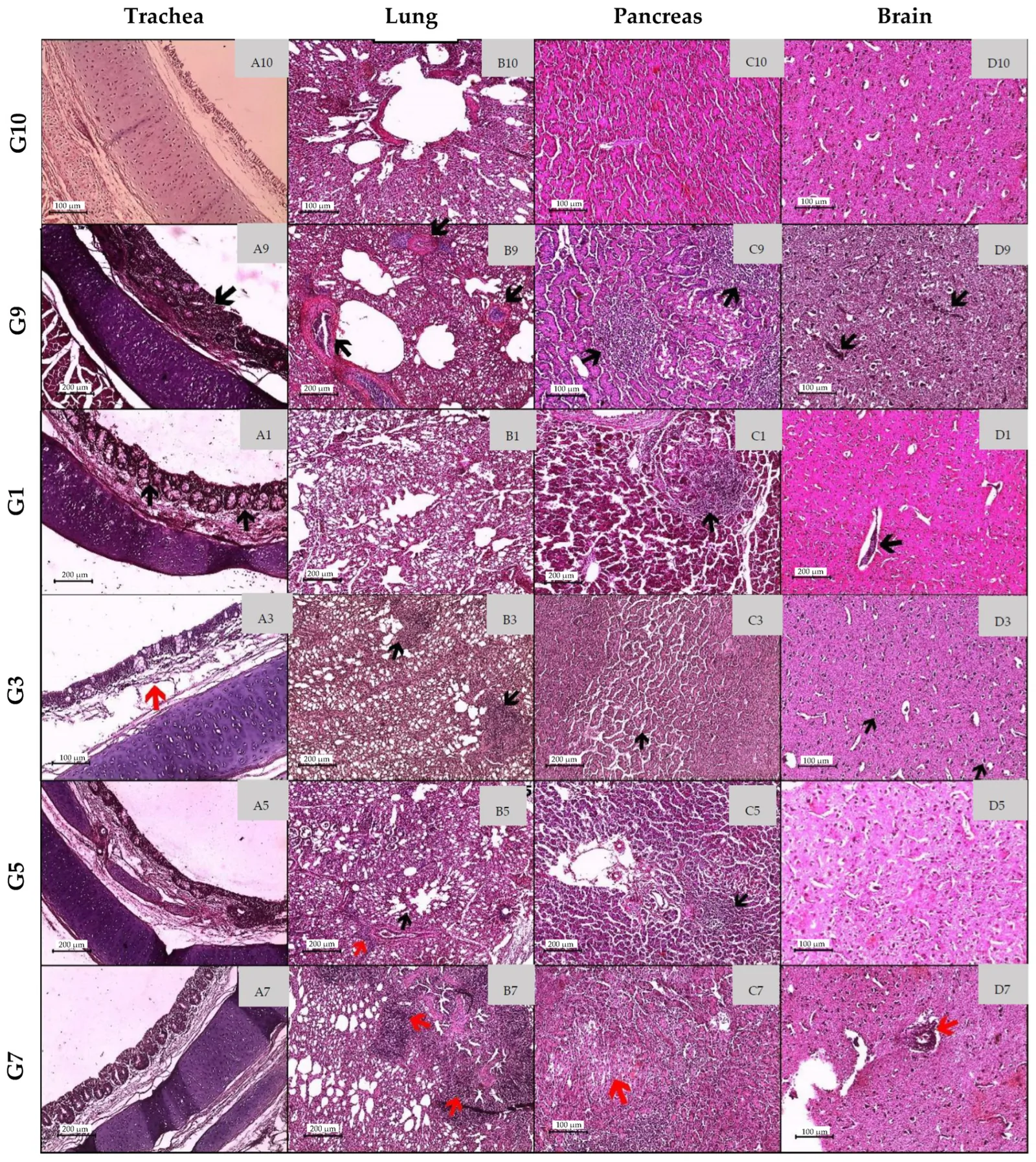
Photomicrographs of the trachea, lung, pancreas, and brain, respectively, of commercial ducks experimentally challenged with highly pathogenic avian influenza virus (H5N1, clade 2.3.4.4b). The representative photomicrographs are presented for the challenged groups (G1, G3, G5, G7), positive control (G9), and negative control (G10). Group 10 (G10, negative control, A10–D10): Histological architecture was nearly normal without significant pathological alterations (scale bar, 100, 200 µm). Group 9 (G9; positive control, A9–D9): +ve control; in (A9), severe pathological changes can be observed, including tracheal epithelial degeneration and desquamation with inflammatory cell infiltration. The trachea shows marked pulmonary congestion and severe tracheitis with prominent thickening of the lining epithelium with massive lymphocytic cell infiltration (black arrow); in (B9), the lung shows marked pulmonary congestion, pneumonia with marked vasculitis infiltrated with massive lymphocytic cell infiltration within the lung parenchyma (black arrow). In (C9), the pancreas shows multifocal necrotic areas infiltrated with massive lymphocytic cell infiltration (arrow). In (D9), the brain shows lymphocytic cell infiltration (black arrow), perivascular cuffing, focal gliosis, and encephalomalacia (scale bar, 100, 200 µm). Group 1 (G1; A1–D1): ValleyVac Avian Flu H5 plus vaccine; in (A1), mild lesions including focal tracheal epithelial disruption with inflammatory infiltration, and the trachea shows goblet cell activation (black arrow) with mild lymphocytic cell infiltration. In (B1), the lung shows a mild pulmonary inflammatory reaction and mild multifocal lymphocytic cell infiltration. In (C1), the pancreas shows mild pancreatic hemorrhages localized to pancreatic inflammatory foci. In (D1), the brain shows mildly congested cerebral blood vessels (black arrow) (scale bar, 100, 200 µm). Group 3 (G3; A3–D3): MEFLUVAC™ H5 PLUS 8 vaccine; in (A3), mild histopathological alterations to the trachea show tracheitis with mild submucosal edema (red arrow); in (B3), the lung shows mild multifocal lymphocytic cell infiltration (black arrow) within the parenchyma. In (C3), the pancreas shows mild pancreatic edema (black arrow). In (D3), mild perineuronal edema and gliosis can be observed in the brain (black arrow) (scale bar, 100, 200 µm). Group 5 (G5; A5–D5): the SERVAC Flu H5N1 vaccine. In (A5), the trachea shows severe submucosal edema with lymphocytic cell infiltration; in (B5), the lung shows pneumonia with multifocal lymphocytic cell infiltration (red arrow) and tertiary bronchi (arrow). In (C5), the pancreas shows moderate pancreatitis with congested blood vessels, hemorrhages, and focal lymphocytic cell infiltration (black arrow). In (D5), the brain shows encephalomalacia and gliosis (scale bar, 100, 200 µm). Group 7 (G7; A7–D7): Sinder Fluvac vaccine; in (A7), the trachea shows tracheitis with submucosal edema and goblet cell activation. In (B7), the lung shows severe diffuse pneumonia with multifocal lymphocytic cell infiltration. In (C7), the pancreas shows a wide necrotic area infiltrated with massive lymphocytic cells (red arrow). In (D7), the brain shows perivascular cuffing (red arrow), inflammatory changes, and gliosis (scale bar, 100, 200 µm). Representative photomicrographs were performed at different magnifications to best illustrate the histopathological changes. Images with a 100 µm scale bar provide an overview of tissue architecture, whereas those with a 200 µm scale bar highlight specific microscopic lesions at higher magnification.

Histopathological examination of the examined groups collected from the trachea, lung, pancreas, and brain revealed markedly variable degrees of tissue alterations among all investigated organs following AIV challenge. The negative control group (G 10) exhibited normal histological structure in all examined organs without significant pathological alterations (Figure 4, A10–D10). In contrast, the most extensive and severe pathological lesions consistent with systemic avian influenza virus infection were observed in the positive control group (G9). The brain showed multiple ischemic capillaries, perivascular cuffing, and diffuse vacuolation. These findings were accompanied by multifocal pancreatic necrosis with marked lymphocytic infiltration, moderate pneumonia with vascular congestion and vasculitis in the lungs, and moderate tracheitis characterized by extensive lymphocytic infiltration, goblet cell activation, and submucosal edema (Figure 4, A9–D9). Group 1 (G1) exhibited mild histopathological alterations, including congested cerebral blood vessels without inflammatory damage and scattered perineuronal edema, pancreatitis with prominent lymphocytic infiltration and edema, and multifocal inflammatory cell infiltration within the pulmonary parenchyma. In this group, the trachea showed mild tracheitis with goblet cell hyperplasia and lymphocytic infiltration. Among the vaccinated groups, there are varying degrees of protection in Group 3 (Figure 4, A1–D1). Similarly, Group 3 (G3) demonstrated mild to moderate lesions compared with the positive control. The trachea exhibited tracheitis associated with submucosal edema. Most diffuse pneumonia with lymphocytic infiltration in the pulmonary tissue and tertiary bronchi was observed, and edema was limited to pancreatic lesions. Although the brain lesions were comparatively milder, they were limited to perineuronal edema and vacuolation (Figure 4, A3–D3). On the other hand, Group 5 (G5) displayed closely moderate histopathological changes. The pancreatic tissues exhibited moderate pancreatitis with vascular congestion and focal inflammatory infiltrates. The lung showed limited pulmonary congestion, pneumonia, and inflammatory infiltration. Diffuse lymphocytic infiltration with severe submucosal edema in the trachea. Brain gliosis and encephalomalacia were observed (Figure 4, A5–D5). A comparable severity was recorded in (G7), which showed pronounced encephalomalacia, gliosis, perivascular cuffing, and vascular congestion in the brain, along with evident multifocal necrotic areas in the pancreas, pneumonia with lymphocytic aggregation in the lungs, and marked inflammatory infiltration in the trachea (Figure 4, A7–D7). Collectively, the severity of histopathological lesions among the studied groups (G5 and G7) may suggest a partial protective effect against tissue damage, whereas the positive control (G9) showed the highest degree of tissue damage and pathological alterations.

## 4. Discussion

In 1996, the HPAI-H5N1 initially emerged in the Chinese poultry sector and was subsequently transmitted across multiple continents via migratory birds’ flyways, causing devastating disease outbreaks in poultry flocks and humans [47,48,49]. In 2020, HPAI-H5N1 strains belonging to clade 2.3.4.4b became the most predominant pathogenic subtype reported in both domestic and wild birds, causing significant damage to wildlife and the poultry industry [21,50]. Despite intensive routine vaccination, Egypt has remained endemic for AIV since February 2006, causing extensive outbreaks in poultry [51]. More recently, the newly emerged H5N1 of clade 2.3.4.4 was introduced into Egypt through migratory waterfowl in late 2021 and is currently co-circulating with other H5 viruses (H5N8 subtypes) of clade 2.3.4.4, disseminating novel AIVs in the Egyptian field [52]. As HPAI becomes a year-round threat, many countries, including Egypt, are weighing options for using vaccination regimes against the viral infection [9]. Thus, this situation underscores the need for efficient vaccines that not only suppress clinical disease but also reduce virus replication and viral shedding. In this context, this study assessed the protective efficacy of AIV-inactivated commercial H5 vaccines against a contemporary circulating H5N1-AIV strain by evaluating clinical findings, histopathology, HI tests, and AIV shedding detection. This current vaccination protocol was experimentally applied in ducks to elucidate protection against the local strain challenge AIV and optimize vaccination strategies.

Due to the endemic status of AIV in Egypt, the national control programs emphasize the use of commercial inactivated vaccines that are genetically and antigenically matched to the current circulating isolates in the field to maximize protective efficacy and minimize virus shedding [53,54]. To the best of our knowledge, this is the first comprehensive assessment in Egypt that investigates the immunogenicity of commercially available inactivated vaccines in ducks against recently circulating H5 *avian influenza viruses*. HPAI-H5 continues to evolve rapidly, creating a major challenge for vaccination programs, particularly in domestic ducks, which play a crucial epidemiological role in the maintenance and dissemination of the virus. Unlike chickens, ducks can survive HPAIV infection while viral shedding still occurs, facilitating silent transmission to susceptible poultry populations and potentially contributing to the emergence of new strains [9,44,55]. Consequently, the current results provide valuable findings on AIV vaccination strategies against AIV-H5N1 in ducks in Egypt. Collectively, no obvious systemic clinical abnormalities were associated with AIV vaccination. In (G9), the nonvaccinated challenged ducks exhibited mild manifestations, and their clinical status worsened from 4 DPC to 10 DPC. These clinical observations are consistent with previous investigations demonstrating that HPAI H5N1 infection in Pekin ducks can lead to widespread viral distribution, resulting in systemic pathological lesions affecting the respiratory, digestive, nervous, and pancreatic tissues [43]. In contrast, ducks vaccinated with ValleyVac Avian Flu H5 plus (H5N1 of clade 2.2.1.2 and reassortant H5N8 of clade 2.3.4.4b) and MEFLUVAC™ H5 PLUS 8 (H5N1 of Clade 2.2.1.2 and Clade 2.2.1.1, and H5N8 of Clade 2.3.4.4b) remained clinically healthy with no pathological lesions, indicating that the vaccine restricted tissue tropism and viral replication. Mild lesions and partial survival were observed in the SERVAC Flu H5N1 and Sinder Fluvac vaccinated groups. These findings are consistent with those from earlier studies [9,38,56]. Additionally, several studies have suggested that suboptimal vaccines may protect against severe disease while allowing limited virus replication in target organs, which may maintain virus circulation within vaccinated flocks [44].

Specifically, the present study demonstrated that all four commercial H5 vaccines provided a degree of protection against challenge with the Egyptian H5N1 clade 2.3.4.4b isolate; however, considerable differences in immunogenicity and protective efficacy were observed. In this experiment, the ValleyVac Avian Flu H5 plus and MEFLUVAC^TM^ H5 PLUS 8 vaccines achieved complete protection against mortality, whereas the SERVAC Flu H5N1 and Sinder Fluvac vaccines provided partial protection. The superior immunogenicity and protective efficacy observed for the ValleyVac Avian Flu H5 plus and MEFLUVAC™ H5 PLUS 8 vaccines are consistent with their reported inclusion of more recently updated H5 antigens, including clade 2.3.4.4-related strains. Although direct molecular comparison between the commercial vaccine seed strains and the challenge virus was not included, our findings are in agreement with previous studies demonstrating that vaccines antigenically closer to circulating field viruses generally induce stronger immune responses and improved protective efficacy [25,57]. Therefore, antigenic relatedness is proposed as a reasonable explanation for the observed differences in vaccine performance. The superior protection induced by ValleyVac Avian Flu H5 plus and MEFLUVAC™ H5 PLUS 8 is likely associated with the presence of clade 2.3.4.4b-related antigens and reverse-genetics-derived vaccine strains. The reverse-genetics method enables the production of safe, high-growth vaccine viruses with updated hemagglutinin antigens, thus improving immunogenicity and cross-protective efficacy against contemporary H5 viruses [44]. The ValleyVac Avian Flu H5 plus vaccine contains reassortant H5N8 and H5N1 subtypes, whereas MEFLUVAC™ H5 PLUS 8 vaccine contains a trivalent isolate formulation including an H5N8 strain belonging to clade 2.3.4.4b. In contrast, the SERVAC Flu H5N1 vaccine is only based on the clade 2.2.1.1 strain, whereas the Sinder Fluvac vaccine contains Re-6 and Re-8 strains that belong to genetically different H5 lineages. Previous studies have demonstrated that small antigenic differences between commercial vaccines and field strains may reduce vaccine efficacy, particularly in terms of virus replication and shedding suppression [36,44]. Similarly, [9,58] demonstrated that H5vaccines of clade 2.3.4.4b provided superior protection in domestic ducks compared with vaccines based on heterologous H5 viruses. Clinically, ducks vaccinated with ValleyVac Avian Flu H5 plus and MEFLUVAC™ H5 PLUS 8 showed 100% survival following challenge, whereas the SERVAC Flu H5N1 and Sinder Fluvac groups showed mortality despite vaccination. Furthermore, classical H5N1 strains cause rapid 100% mortality in chicken flocks; mortality rates of 50–70% in domestic ducks are typical for clade 2.3.4.4b HPAI isolates [59,60]. Thus, the mortality observed in the unvaccinated challenged control group (66.7%) is consistent with the known variability of HPAI virus pathogenicity in domestic ducks. Pekin ducks frequently exhibit variable mortality following HPAI H5N1 infection, influenced by viral strain, host age, inoculation dose and route, host innate immune responses, and genetic susceptibility [6,61]. Thus, the complete inhibition of mortality and clinical disease in groups (G1) and (G3) underscores the robust protective efficacy of these clade-matched formulations. Consequently, vaccine efficacy was interpreted using multiple outcome measures, including survival, clinical disease, viral shedding, and pathological findings, rather than mortality alone. These findings indicate marked differences in vaccine-induced protection. The present study did not directly evaluate the antigenic relationship between the commercial vaccine seed strains and the challenge virus; therefore, the observed differences in protection cannot be attributed solely to antigenic matching. Nevertheless, previous studies have consistently demonstrated that antigenic relatedness between vaccine strains and circulating field viruses is an important determinant of vaccine efficacy [52]. Accordingly, antigenic relatedness represents one reasonable explanation for the superior performance of the ValleyVac Avian Flu H5 plus and MEFLUVAC™ H5 PLUS 8 vaccines, together with other factors such as vaccine formulation, antigen content, adjuvant characteristics, and the quality of the immune response. Similar findings were reported by [44], which observed that an inactivated recombinant vaccine (clade 2.3.4.4b) induced broad cross-protection against cocirculating H5Nx viruses. Moreover, [40] demonstrated that vaccines made with updated H5 antigens significantly improved the protection against recently circulating H5N8 viruses in Egyptian poultry. The lower protection induced by the two vaccines in the present study may be due to the ongoing genetic and antigenic drift of Egyptian H5 viruses.

The humoral immune kinetics observed in the non-challenged parallel groups (G2, G4, G6, and G8) closely aligned with the protection levels observed in their corresponding challenged cohorts (G1, G3, G5, and G7). Consequently, these results were used to interpret the relationship between vaccine-induced humoral immunity and the subsequent protection observed after the challenge. While blood sampling was not collected directly from the challenge groups to prevent handling-induced physiological stress, hematoma formation, and potential corticosteroid-driven immunosuppression prior to HPAI inoculation, blood sampling was omitted in the challenge groups (G1, G3, G5, and G7). Given that parallel groups were randomized from the same batch, housed identically, maintained under the same experimental conditions, and vaccinated using the same vaccine lots with similar vaccination protocols. These subsequent findings agree with [43,62,63,64]. Surprisingly, the humoral immune response closely paralleled the observed level of protection. ValleyVac Avian Flu H5 plus and MEFLUVAC™ H5 PLUS 8 vaccines induced significantly higher HI antibody titers than SERVAC Flu H5N1 and Sinder Fluvac vaccines throughout the experimental period. The earlier protection level and seroconversion observed with these vaccines may indicate a more effective antigen presentation and improved stimulation of B-cell responses, possibly resulting from better antigenic compatibility and optimized vaccine formulation [52]. According to the WOAH guidelines [65], HI antibody titers exceeding 5 log_2_ are associated with protection against mortality, whereas higher antibody levels reduce virus replication and shedding [66]. Thus, the HI titers induced by the ValleyVac and MEFLUVAC vaccines contributed to their superior protective efficacy. The higher HI antibody titers obtained with the homologous local antigen (Newvalley-3-H5N1-2024, clade 2.3.4.4b) are consistent with previous reports demonstrating that homologous antigens elicit greater HI titers than heterologous H5 antigens because of their closer antigenic relationship to the circulating field strains [24,67]. Likewise, the higher antibody titers of the homologous antigen, together with the superior survival and reduced viral shedding observed after homologous challenge, highlight the importance of antigenic matching for accurate vaccine evaluation and may further support the need to update vaccine seed strains according to contemporary molecular surveillance data. In endemic countries such as Egypt, where H5 viruses undergo continuous evolution, dependence on older reference antigens may underestimate vaccine-induced immunity and hinder vaccine evaluation.

In addition to the HI assay using homologous antigen, antibody responses were evaluated using a standardized heterologous H5N8 reference antigen (A/Chicken/Netherlands/EMC-3/2014), which belongs to HA clade 2.3.4.4c, whereas the challenge virus (Newvalley-3-H5N1-2024) belongs to clade 2.3.4.4b. The lower HI titers observed against the heterologous clade 2.3.4.4c antigen across all vaccinated groups may demonstrate the impact of antigenic drift on antibody binding efficiency. Although both viruses originate from the broader 2.3.4.4 lineage, accumulated amino acid substitutions within the HA globular head may alter antigenic epitopes recognized by neutralizing antibodies, thereby reducing antibody binding affinity and resulting in lower heterologous HI titers. These subsequent findings are inconsistent with [68,69,70,71,72]. Nevertheless, the detection of measurable HI antibodies against the heterologous antigen, especially in ValleyVac Avian Flu H5 plus^®^ and MEFLUVAC^TM^ H5 PLUS 8^®^ vaccines, indicates that vaccination elicited cross-reactive antibodies capable of recognizing and targeting conserved epitopes shared among different H5 clades. Such cross-reactive immunity may contribute to partial cross-protection against antigenically related viruses; however, homologous HI titers remain more effective in protection against the circulating field strain because they more accurately reflect antigenic matching. Thus, in Egypt, where H5N1 viruses are continuously evolving, measuring heterologous HI antibody responses provides valuable information on the breadth of vaccine-induced immunity. Vaccines that only generate high antibody titers against homologous strains may not provide sufficient protection against antigenically distinct field viruses, leaving poultry populations unprotected against newly emerging variants [72,73]. Therefore, the higher heterologous HI titers induced by the ValleyVac Avian Flu H5 plus (G2) and MEFLUVAC™ H5 PLUS 8 (G4) vaccines suggest broader cross-reactive immunity and a greater potential for protection against genetically related H5 variants.

The reduction in viral shedding represents one of the most important objectives of HPAI vaccination because vaccinated birds that survive infection but continue to shed AIV may contribute to silent viral circulation and increase the risk of virus persistence and evolution under field conditions [68,71,73]. As depicted in our results, all vaccinated groups showed significantly lower viral shedding than the unvaccinated challenged controls; however, among vaccines, the significance of reduction differed considerably. Currently, the ValleyVac Avian Flu H5 plus vaccine exhibited the greatest viral load reductions in both tracheal and cloacal shedding at every time point, followed closely by the MEFLUVAC™ H5 PLUS 8 vaccine, whereas the SERVAC Flu H5N1 and Sinder Fluvac vaccines showed higher viral shedding. These findings agree with previous studies demonstrating that commercial vaccines antigenically matched to circulating AIV-clade 2.3.4.4b viruses significantly reduce viral replication and shedding [9,11,56,74]. From an epidemiological perspective, a noteworthy reduction in viral load is crucial in mortality prevention because it limits flock-to-flock transmission and reduces virus persistence and antigenic evolution [75,76]. Generally, viral loads were higher in tracheal swabs than in cloacal swabs during the early post-challenge period, indicating that the respiratory tract was the primary site of viral replication. This observation agrees with previous pathogenesis studies demonstrating that contemporary AIV H5N1-clade 2.3.4.4b efficiently replicates in the upper respiratory tract of ducks before systemic dissemination [21,77]. The progressive reduction in both tracheal and cloacal shedding after vaccination suggests that the vaccine-induced immune response effectively limited viral replication. The absence of detectable virus at several time points in the ValleyVac and MEFLUVAC groups further supports the superior efficacy of these commercial vaccines.

Histopathological examination remains one of the most reliable approaches for evaluating the efficacy of AIV vaccines. Importantly, histopathological findings indicate that vaccination can efficiently reduce AIV-associated tissue damage compared with unvaccinated challenged controls. The degree of protection varied among vaccines, with ValleyVac Avian Flu H5 plus and MEFLUVAC^TM^ H5 PLUS 8 vaccines showing the most effective results. In the positive control group, extensive and severe lesions, including systemic dissemination and pathological lesions, are characteristic of HPAI infection. Furthermore, the marked tracheitis, pneumonia, and pancreatic lesions reflect active AIV replication within the host’s multisystem. Similar lesions have been reported in experimentally infected birds challenged with HPAI [27,40,46]. Notable findings in (G6) included encephalomalacia, gliosis, vascular congestion in the brain, multifocal necrotic areas in the pancreas, pneumonia with lymphocytic aggregation in the lungs, and marked inflammatory infiltration in the trachea. These lesions were parallel with those in [27,40,46]. Pancreatic lesions were evident in HPAI systemic infection, resulting in high viral loads, increased disease severity, and lower protective efficacy. Among the vaccinated groups, (G3) and (G4) provided the best histopathological protection with limited lesions. These mild histopathological alterations indicate restricted systemic spread of the challenge virus. The presence of mild-to-moderate lesions in vaccinated ducks indicates that although vaccination reduced disease severity, it may not completely prevent viral replication. These findings are consistent with those of previous studies demonstrating that AIV vaccination often decreases clinical signs, mortality rate, and tissue damage but does not always achieve complete immunity [22,60,62,78]. Therefore, vaccination programs should be integrated with comprehensive surveillance and biosecurity approaches to achieve disease control in duck populations.

The findings of the present study are consistent with the successful avian influenza vaccination strategy implemented in China, where continuous nationwide virological surveillance is closely integrated with periodic updating of H5 vaccine seed strains in response to antigenic evolution of circulating viruses [79,80]. Rather than relying on fixed vaccine formulations, China routinely performs genetic and antigenic characterization of newly emerging H5 viruses and rapidly replaces vaccine seed strains using reverse-genetics technology whenever significant antigenic drift is detected. This dynamic vaccine iteration strategy has maintained high levels of protection against successive H5 virus lineages, including clade 2.3.4.4b H5N1 viruses, as well as reducing viral shedding and field outbreaks in both chickens and ducks [79,80,81]. Our findings similarly demonstrate that vaccines containing more recently updated H5 seed strains induced stronger homologous antibody responses, complete protection against mortality, and greater suppression of viral shedding than vaccines based on older antigenic variants. Although the present study provides a comprehensive comparative evaluation of four commercially available H5 vaccines under standardized laboratory conditions, it was limited to a single experimental challenge using one contemporary Egyptian HPAI H5N1 clade 2.3.4.4b isolate in Pekin ducks. Future studies should include independent challenge experiments, additional duck breeds, recently emerging H5 variants, longer post-vaccination observation periods, and field-based evaluations to further validate the protective efficacy and durability of these commercial vaccines under diverse epidemiological conditions.

Collectively, the present study provides practical evidence for optimizing HPAI H5 vaccination strategies in duck production systems in Egypt. Although all four commercial vaccines induced measurable humoral immune responses, their protective efficacy differed considerably when evaluated against a contemporary Egyptian H5N1 clade 2.3.4.4b challenge virus. Vaccines incorporating antigenically updated reverse-genetics-derived seed strains produced superior homologous antibody responses, complete protection against mortality, and markedly reduced viral shedding compared with vaccines based on older field isolates or antigenically distant strains. These findings highlight the importance of maintaining antigenic compatibility between vaccine seed viruses and circulating field viruses. Given the continuous evolution of HPAI H5 viruses in Egypt, periodic updating of commercial vaccine seed strains and routine monitoring of vaccine–field virus antigenic relationships should be considered essential components of national avian influenza control programs.

## 5. Conclusions

This study emphasizes the crucial role of an efficient vaccination regime in avian influenza control strategies in Egypt and provides baseline data from Pekin ducks to support the development of optimized vaccination programs. This study demonstrates marked differences in the protective efficacy of commercially available inactivated H5 vaccines against a contemporary Egyptian HPAI H5N1 isolate (Newvalley-3-H5N1-2024, clade 2.3.4.4b) in Pekin ducks. The ValleyVac Avian Flu H5 plus and MEFLUVAC™ H5 PLUS 8 vaccines elicited high humoral immune responses, providing complete clinical protection against mortality and significantly reduced tracheal and cloacal viral shedding following challenge. In contrast, SERVAC Flu H5N1 and the Sinder Fluvac vaccines revealed only partial protection and allowed higher viral replication and shedding. Additionally, these findings highlight the critical role of antigenic compatibility between available commercial vaccines and circulating field strains in achieving optimal vaccine efficacy and efficient clinical protection. The results also highlight the epidemiological importance of reducing viral shedding in ducks, which may act as silent carriers and contribute to HPAI virus persistence and spread. In Egypt, continuous molecular surveillance, regular antigenic characterization of emerging isolates, and periodic updating of vaccine seed strains should be integrated into national avian influenza control programs. Such an optimized vaccination regime combined with strict biosecurity measures and ongoing genomic monitoring will be essential for limiting AIV circulation, reducing economic losses, maintaining adequate field protection, and minimizing the risk of further evolution and dissemination of HPAI H5N1 viruses in commercial duck populations.

## Figures and Tables

**Table 1:** H5 commercial vaccines used for immunizing Pekin ducks against HPAI-H5N1 in Egypt.

Vaccine Trade Name	Virus Strain	Virus Lineage	Batch Number	Manufacturer, Country
ValleyVac Avian Flu H5 plus	- Inactivated AIV, H5N1 reassortant strain RG A/chicken/Egypt/D10552B/2015 (H5N1) and subtype H5N8 reassortant strain RG A/green-winged teal/Egypt/877/2016 (H5N8), emulsified in an oil adjuvant. - Both strains were constructed as reverse genetics (RG) 6:2 reassortant vaccine seed viruses following the standard approach for HPAI vaccine development. The manufacturer does not publicly specify the internal gene backbone.	- Clade 2.2.1.2 - Clade 2.3.4.4b	242005003	The Egyptian Company for the Biological and Pharmaceutical Industries (Vaccine Valley), Giza, Egypt
SERVAC Flu H5N1	Influenza A/Chicken/Egypt/M2583D/2010 (H5N1) [conventional (non-RG) wild-type isolate without reassortment].	- Clade 2.2.1.1	23032	Veterinary Serum and Vaccine Research Institute (VSVRI), Abbassia, Cairo, Egypt
MEFLUVAC^TM^ H5 PLUS 8	- Inactivated AIV, H5N1 subtype [A/Chicken/Egypt/RG-173 CAL/2017(H5N1)]. - Inactivated avian influenza H5N1 subtype [RgA/CK/Egypt/ME1010/2016(H5N1)]. - Inactivated AIV, H5N8 subtype [RgA/chicken/ME-2018/H5N8]. - All three seed strains generated using plasmid-based reverse-genetics technology.	- Clade 2.2.1.2 - Clade 2.2.1.1 - Clade 2.3.4.4b	2401210101	Middle East for Vaccines Cairo, (MEVAC, Cairo, Egypt).
Sinder Fluvac	Bivalent H5N1 inactivated vaccine containing Re-6 + Re-8 H5N1 subtype strains: - RG reassortant strains Re-6 strain: (A/duck/Guangdong/S1322/2010 (H5N1). - Re-8 strain: A/chicken/Guizhou/4/2013 (H5N1). - Both Re-6 and Re-8 strains were constructed via 6:2 plasmid reverse genetics utilizing the PR8 internal gene framework according to the manufacturer.	- Re-6 strain → clade 2.3.2.1b - Re-8 strain → clade 2.3.4.4g	20230401	Shandong Sinder Animal Vaccine Co., Ltd., Zhucheng, China

**Table 2:** Experimental vaccination design using four commercial vaccines against local isolate, HPAI-H5N1 of clade 2.3.4.4b.

Group	Vaccination Regime	Number of Ducks in the Study	Age at Vaccination		Number of Ducks Challenged
			On Day 10		
1	ValleyVac Avian Flu H5 plus	15 in each group	10 days of age		15
2	ValleyVac Avian Flu H5 plus *		10 days of age		Non
3	MEFLUVAC^TM^ H5 PLUS 8		10 days of age		15
4	MEFLUVAC^TM^ H5 PLUS 8 *		10 days of age		Non
5	SERVAC Flu H5N1		10 days of age		15
6	SERVAC Flu H5N1 *		10 days of age		Non
7	Sinder Fluvac		10 days of age		15
8	Sinder Fluvac *		10 days of age		Non
9	Positive control		The nonvaccinated challenged group		15
10	Negative control		The nonvaccinated and nonchallenged groups		15

* vaccinated non-challenged groups (15 ducks per group).

## Data Availability

Data are available upon request.

## Supplementary Materials

The following supporting information can be downloaded at https://www.mdpi.com/article/10.3390/v18080891/s1. Figure S1: Hemagglutination inhibition antibody titers line graph by HI assay in ducks (G2, G4, G6, and G8) vaccinated with AIV commercial vaccines via the homologous challenge virus antigen (A) and heterologous reference H5 antigen (B) at 7-, 14-, 21-, and 28 DPV. Data are expressed as group mean ± SD titer from 5 ducks per group. Statistical Variations between groups were determined via Tukey’s post-hoc multiple comparisons test, with significance statistically established at *p*-value ≤ 0.05; Table S1: Statistical analysis of histopathological alterations in different organs of experimentally challenged ducks following vaccination; Table S2: HI titers assessment (log2) in ducks (G2, G4, G6, and G8) vaccinated with four inactivated vaccines by HI assay using the homologous challenge virus antigen (Newvalley-3-H5N1-2024 of clade 2.3.4.4b) at weekly intervals (7, 14, 21, and 28) DPV; Table S3: HI titers assessment (log2) in ducks (G2, G4, G6, and G8) vaccinated with four inactivated vaccines by HI assay using the heterologous challenge virus antigen (Newvalley-3-H5N1-2024 of clade 2.3.4.4b) at weekly intervals (7, 14, 21, and 28) DPV.
