# Supplementary material for: Protective Efficacy Evaluation of Various Inactivated Vaccines Against the Newly Circulated Highly Pathogenic Avian Influenza Virus H5N1 of Clade 2.3.4.4b in Pekin Ducks
Source: Viruses. 2026 Aug 13;18(8):891. doi: 10.3390/v18080891 (PMC13517388; doi:10.3390/v18080891)
Supplement: Supplementary file 1 [file viruses-18-00891-s001.zip › viruses-4447848-supplementary.pdf]

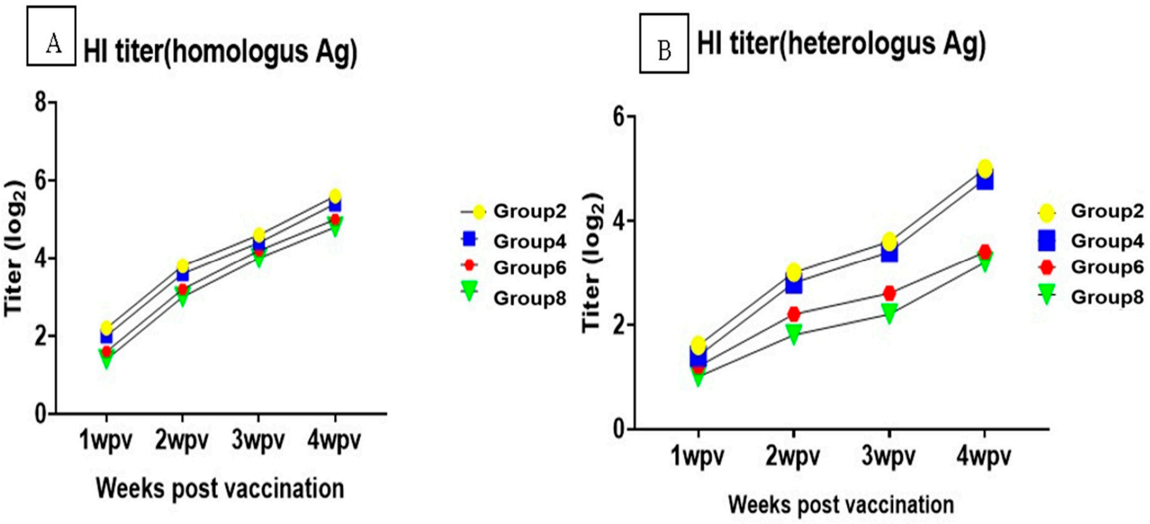

Figure S1: Hemagglutination inhibition antibody titers line graph by HI assay in ducks (G2, G4, G6, and G8) vaccinated with AIV commercial vaccines via the homologous challenge virus antigen (A) and heterologous reference H5 antigen (B) at 7-, 14-, 21-, and 28 DPV. Data are expressed as group mean ± SD titer from 5 ducks per group. Statistical Variations between groups were determined via Tukey's post-hoc multiple comparisons test, with significance statistically established at P-value ≤ 0.05.

Table S1: Statistical analysis of histopathological alterations in different organs of experimentally challenged ducks following vaccination.

	GROUP				
**ORGAN**	**POSITIVE CONTROL**	**VACCINE VAALEY**	**MEVAC**	**SERVAC**	**CHINESE**
**LUNG**					
CONGESTION	2.75 ± 0.50^{a}	2.5 ± 0.58^{a}	2.75 ± 0.50^{a}	2.5 ± 0.58^{a}	2.25 ± 0.50^{a}
LYMPHOCYTIC INFILTRATION	3.0 ± 0.0^{a}	1.75 ± 0.50^{b}	2.5 ± 0.58^{a}	2.5 ± 0.58^{a}	2.75 ± 0.50^{a}
NECROSIS	2.75 ± 0.50^{a}	2.25 ± 0.96^{a}	2.5 ± 0.58^{ab}	2.0 ± 0.82^{ab}	2.0 ± 0.82^{a}
**TRACHEA**					
CONGESTION	2.25 ± 0.50^{a}	2.0 ± 0.0^{a}	1.50 ± 0.58^{a}	1.75 ± 0.50^{a}	1.75 ± 0.50^{a}
SUBMUCOSAL EDEM	2.75 ± 0.50^{ab}	1.25 ± 0.50^{c}	1.50 ± 0.58^{bc}	2.5 ± 0.58^{a}	2.25 ± 0.50^{a}
MUCOSAL NECROSIS	1.50 ± 0.58^{a}	0.50 ± 0.58^{b}	1.25 ± 0.50^{ab}	1.25 ± 0.50^{ab}	0.75 ± 0.50^{ab}
GOBLET CELLS ACTIVATION	3.0 ± 0.0^{ab}	2.25 ± 0.50^{bc}	2.0 ± 0.0^{c}	2.5 ± 0.58^{abc}	2.75 ± 0.50^{a}
**PANCREASE**					
EDEMA	1.75 ± 0.50^{a}	1.75 ± 0.98^{a}	1.0 ± 0^{a}	1.50 ± 0.58^{a}	1.50 ± 0.58^{a}
LYMPHOCYTIC INFILTRATION	2.75 ± 0.50^{a}	1.50 ± 0.58^{b}	1.50 ± 0.58^{b}	1.75 ± 0.50^{b}	2.75 ± 0.50^{a}
NECROSIS	2.75 ± 0.50^{a}	2.25 ± 0.96^{a}	0.75 ± 0.50^{b}	1.75 ± 0.50^{a}	2.75 ± 0.50^{a}
**BRAIN**					
PERIVASCULAR CUFFING	1.50 ± 0.58^{a}	0.50 ± 0.58^{b}	0.25 ± 0.50^{b}	0.75 ± 0.96^{b}	2.0 ± 0.0^{a}
CONGESTION	2.5 ± 0.58^{ab}	1.25 ± 0.50^{c}	1.25 ± 0.50^{c}	1.50 ± 0.58^{bc}	2.25 ± 0.50^{a}
GLIOSIS	2.75 ± 0.50^{ab}	1.25 ± 0.50^{c}	1.25 ± 0.50^{c}	2.5 ± 0.58^{bc}	2.5 ± 0.58^{a}

Different small letters indicate significantly different between groups within the same row. Data were expressed as mean ± SD at ($P < 0.05$).

Table S2: HI titers assessment (log2) in ducks (G2, G4, G6, and G8) vaccinated with four inactivated vaccines by HI assay using the homologous challenge virus antigen (Newvalley-3-H5N1-2024 of clade 2.3.4.4b) at weekly intervals (7, 14, 21, and 28) DPV.

Group	1WPV	2WPV	3WPV	4WPV
G2	2.2^{a}	3.8^{a}	4.6^{a}	5.6^{a}
G4	2.0^{ab}	3.6^{a}	4.4^{a}	5.4^{a}
G6	1.6^{ab}	3.2^{ab}	4.2^{a}	5.0^{a}
G8	1.4^{c}	3^{c}	4^{c}	4.8^{c}

Table S3: HI titers assessment (log2) in ducks (G2, G4, G6, and G8) vaccinated with four inactivated vaccines by HI assay using the heterologous challenge virus antigen (Newvalley-3-H5N1-2024 of clade 2.3.4.4b) at weekly intervals (7, 14, 21, and 28) DPV.

Group	1WPV	2WPV	3WPV	4WPV
G2	1.6^{bc}	3^{bc}	3.6^{b}	5^{ab}
G4	1.4^{bc}	2.8^{bc}	3.4^{b}	4.8^{ab}
G6	1.2^{bc}	2.2^{bc}	2.6^{c}	3.4^{bc}
G8	1.0^{c}	1.8^{c}	2.2^{c}	3.2^{c}
